# The Limits on Trypanosomatid Morphological Diversity

**DOI:** 10.1371/journal.pone.0079581

**Published:** 2013-11-19

**Authors:** Richard John Wheeler, Eva Gluenz, Keith Gull

**Affiliations:** 1 Sir William Dunn School of Pathology, University of Oxford, Oxford, United Kingdom; University of Texas Medical School at Houston, United States of America

## Abstract

Cell shape is one, often overlooked, way in which protozoan parasites have adapted to a variety of host and vector environments and directional transmissions between these environments. Consequently, different parasite life cycle stages have characteristic morphologies. Trypanosomatid parasites are an excellent example of this in which large morphological variations between species and life cycle stage occur, despite sharing well-conserved cytoskeletal and membranous structures. Here, using previously published reports in the literature of the morphology of 248 isolates of trypanosomatid species from different hosts, we perform a meta-analysis of the occurrence and limits on morphological diversity of different classes of trypanosomatid morphology (trypomastigote, promastigote, etc.) in the vertebrate bloodstream and invertebrate gut environments. We identified several limits on cell body length, cell body width and flagellum length diversity which can be interpreted as biomechanical limits on the capacity of the cell to attain particular dimensions. These limits differed for morphologies with and without a laterally attached flagellum which we suggest represent two morphological superclasses, the ‘juxtaform’ and ‘liberform’ superclasses. Further limits were identified consistent with a selective pressure from the mechanical properties of the vertebrate bloodstream environment; trypanosomatid size showed limits relative to host erythrocyte dimensions. This is the first comprehensive analysis of the limits of morphological diversity in any protozoan parasite, revealing the morphogenetic constraints and extrinsic selection pressures associated with the full diversity of trypanosomatid morphology.

## Introduction

Protozoan parasite life cycles are often characterised by specialised proliferative and transmissive life cycle stages, each of which represents an adaptation to that host environment (for a replicative stage) or a pre-adaptation to the next host environment and any conditions likely to be encountered during transmission (a transmissive stage). It is often the case that transmissive stages are non-proliferative, meaning a parasite life cycle is often made up of several linked proliferative cycles. Trypanosomatids, which are a diverse order of exclusively parasitic protozoa with a monoxonous life cycle in an insect host or a dixenous life cycle between an invertebrate and vertebrate or plant host, include many excellent examples of this life cycle structure. This family includes the human pathogens *Leishmania* spp., *Trypanosoma brucei* and *Trypanosoma cruzi*.

**Figure 1:**
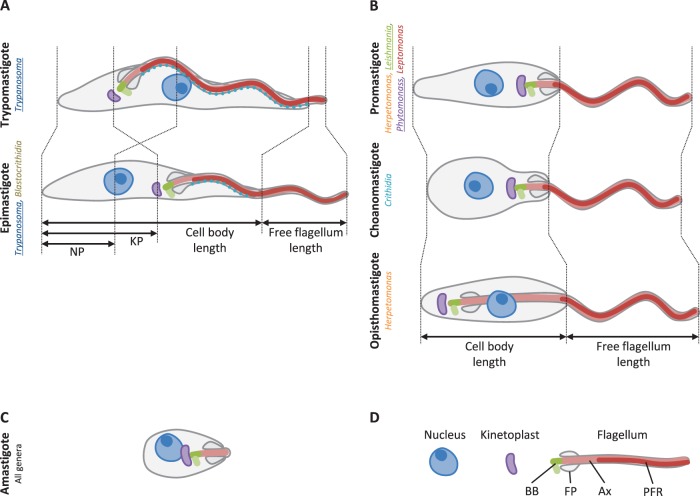
The major morphological classes of trypanosomatids. Diagrams of six common, easily distinguished trypanosomatid morphologies [3]. **A.** Morphologies with a flagellum laterally attached to the cell body. **B.** Morphologies with a free flagellum (no lateral attachment of the flagellum to the cell body extending beyond the flagellar pocket neck). Metrics used to record cell morphology are indicated (cell body length, free flagellum length, kinetoplast–posterior distance (KP) and nucleus–posterior distance (NP)). Genera in which each morphology occurs [28] in are indicated, monophyletic genera [25] are underlined. **C.** Amastigote morphology, which does not have a long, motile, flagellum. **D.** Key. Structures associated with the flagellum (the basal body/pro-basal body pair (BB), flagellar pocket (FP), Axoneme (Ax) and paraflagellar rod (PFR)) are indicated.

Life cycle stage adaptation may incorporate many metabolic, biochemical and cell biological adaptations, including adaptation of cell shape. In trypanosomatids large morphological variation occurs both between life cycle stages and between species, despite great ultrastructural similarity which is universally conserved [1], [2]. This diversity of shape has been catalogued extensively as light microscopy provided the earliest means for classifying these microorganisms, with six major morphological classes commonly defined by the position and depth of the flagellar pocket, flagellum length, and lateral attachment of the flagellum to the cell body (Figure 1) [3]. The function of these cell shapes is largely unknown, although there are examples indicating that correct morphogenesis is vital for pathogenicity [4]. Together these properties mean the trypanosomatids are an excellent model for considering the function of cell shape in a unicellular parasite’s pathogenicity, and how this links to the capacity for morphological change for adaptation to different host environments.

The diversity, and limits on diversity, of the morphologies of life cycle stages of any parasite are likely to have arisen through two biological phenomena. Firstly intrinsic biomechanical constraints arising from the cell organisation (and its growth and division) may limit the range of potential viable cell shapes. Secondly selective pressures from the host environment may limit the range of these viable cell shapes actually observed in different host environment. In qualitative terms if occurrence of particular morphological classes are limited to particular environments it is suggestive of a selection pressure; for example in trypanosomatids a selection for trypomastigotes in the vertebrate bloodstream [1], [5]–[9] and for amastigotes in intracellular life cycle stages [10]–[12] appear to have occurred. In a similar line of reasoning, trypanosomatid morphologies universally have a sub-pellicular microtubule cytoskeleton, flagellum and flagellar pocket, even in immotile amastigotes [10]–[14], indicating their role in basic cell organisation confers a biomechanical constraint. This limit in basic cell organisation is consistent with the well known and diverse roles of the flagellar pocket and associated structures in trypanosomatids, particularly for kinetoplast division [15], [16] and endo and exocytosis [17]–[20] amongst others [21].

We reasoned that more information about constraints and selective pressures acting on trypanosomatid shape could be inferred by analysing limits on trypanosomatid shape and size quantitatively, and that this could be done most effectively if we considered multiple classes of trypanosomatid morphology in combination. Whether any particular limit in morphological diversity arises from a constraint or a selective pressure could be determined by analysing multiple classes of morphology in different environments. Constraints universal to all trypanosomatids will give rise to limits to cell shape in all species and classes, while constraints restricted to only particular morphological classes will give rise to limits to cell shape within only those classes. Limits in cell shape observed in trypanosomatids of a particular morphological class in one host environment but not another may have arisen through host selective pressures.

In order to detect these potential constraints or selective pressures, we performed a literature-based meta-analysis of trypanosomatid morphology in a wide range of species. Morphometric data from 248 isolates of trypanosomatids from the vertebrate bloodstream or invertebrate host were extracted from the literature to analyse cell length, cell width, flagellum length and flagellar pocket positioning. We established the concept of two morphological superclasses, the juxtaforms (trypomastigotes and epimastigotes) and liberforms (promastigotes, choanomastigotes and opisthomastigotes) on the bases of the phylogeny of the species in which they occur and the incapacity of trypanosomatids to transition between these two superclasses in the life cycle as a basis for analysing these morphometric data. The correlations of quantitative morphological measures were used to analyse whether morphological superclasses are a valid concept and determine limits on morphological variation which may be associated with constraints intrinsic to the juxtaforms and liberforms. In juxtaforms, morphological diversity, in combination with correlation of trypanosomatid shape with host erythrocyte sizes, was used to analyse limits on morphological variation in the blood environment. Some of these limits may be associated with selective pressures from the bloodstream and we determine which are consistent with proposed functions of cell shape and motility in *T. brucei*. We then discuss which mechanisms may apply constraints and selection pressures consistent with our observations which may have driven the evolution of trypanosomatid morphological diversity.

## Results

In order to perform the meta-analysis of limits to trypanosomatid cell shape associated with constraints or selective pressures we generated a database of previously published trypanosomatid morphometric data for motile life cycle stages of many different species from different vertebrate and invertebrate hosts (Table S1). The literature describing new species or isolates was systematically surveyed, guided by existing indices of trypanosomes [22], [23] and other trypanosomatids [23], [24], and supplemented by database searches for more recently described species and isolates. This yielded approximately 250 references and each was screened to determine if it included suitable morphometric data. Briefly (for more detail see Materials and Methods), if morphology measurements were available and taken from a sample immediately derived from a host or axenic cultures immediately derived from an isolate from a host, and data were presented as representative of the complete range of trypanosomatid dimensions present in that life cycle stage then it was judged to be of sufficient quality for inclusion in the morphometry database. Cell body length and width, free and total flagellum length and kinetoplast to posterior distance and the ranges of each of these measures (or the available subset) were recorded. All morphological analyses, unless otherwise indicated, are derived from these data which is the first quantitative survey of trypanosomatid shape of this magnitude. Data collection was focused on motile life cycle stages and identified examples of *Trypanosoma* in the bloodstream and invertebrate hosts, and *Blastocrithidia, Leishmania, Phytomonas, Leptomonas, Crithidia, Herpetomonas, Strigomonas, Angomonas and Paratrypanosoma* in invertebrate hosts. This represents coverage of the *Trypanosoma, Phytomonas,* Leishmaniinae, *Blastocrithidia, Herpetomonas* and the endosymbiont-bearing clades [25] and the newly-identified *Paratrypanosoma* genus which is the most basal known trypanosomatid lineage [26]. Descriptions of *Leishmania, Phytomonas* and *Trypanosoma* morphology in the invertebrate host were comparatively rare, reports were dominated by those of amastigotes from vertebrates, promastigotes from plants and trypomastigotes from vertebrates respectively.

**Figure 2:**
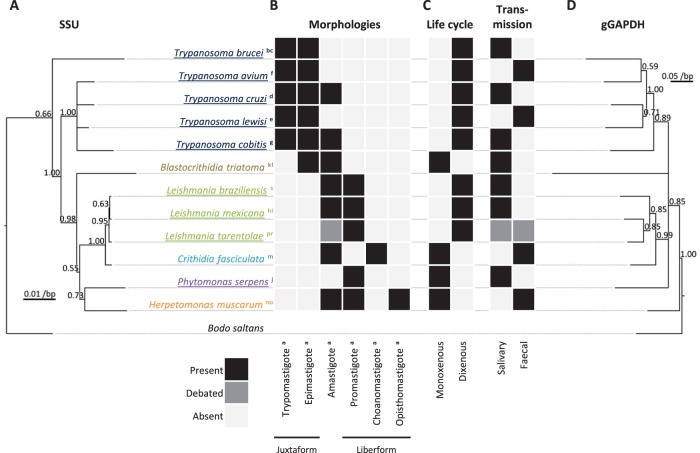
Trypanosomatid morphology, life cycle and phylogeny are indicative of two morphological superclasses. **A.** Phylogeny of 12 representative trypanosomatids inferred from the small subunit (SSU) rRNA gene sequence, rooted with the outgroup *B. saltans.* Values at nodes indicate bootstrap support. The apparent paraphyly of *Trypanosoma* is a well documented example of a long branch attraction artefact [212]. **B.** Morphological classes attained though the 12 trypanosomatid life cycles. ^a^ [3], ^b^ [1], ^c^ [8], ^d^ [6], ^e^ [9], ^f^ [5], ^g^ [7], ^h^ [213], ^i^ [36], ^j^ [195], ^k^ [214], ^l^ [215], ^m^ [216], ^n^ [217], ^o^ [187], ^p^ [218], ^r^ [219], ^s^ [213]. **C.** Life cycle type and transmission route from the insect host in the 12 trypanosomatid life cycles. Relevance of the *L. tarentolae* amastigote in the life cycle [220], [221] and the transmission pathway [213] are debated. **D.** Phylogeny of the 12 trypanosomatids inferred from the glycosomal glyceraldehyde-3-phosphate dehydrogenase (gGAPDH), rooted with the outgroup *B. saltans.* Values at nodes indicate bootstrap support.

For analysis these morphometric data required placement into morphological classes. There are well established morphological classes (trypomastigote, epimastigote, promastigote, choanomastigote, opisthomastigote and amastigote) for trypanosomatids which have historically been used to define the genera (Figure 1) [24], [27], [28]. Several of these genera have since been shown to be paraphyletic [25] indicating this degree of morphological subclassification is taxonomically deceptive. We therefore aimed to superclassify morphologies in a more biological relevant way guided by the phylogeny of trypanosomatids and the morphological transitions they can undergo through the life cycle. A comprehensive analysis of trypanosomatid morphological class occurrence by phylogeny would have been desirable, however there is little overlap between species description by morphology and by genetic data. Therefore we instead focused on fewer high quality descriptions of species morphology through the whole life cycle where both small subunit (SSU) rRNA and glycosomal glyceraldehydephosphate dehydrogenase (gGAPDH) sequence data (the most commonly sequenced genes for species identification and phylogenetic analysis of trypanosomatids) were available in GenBank [29]. This analysis revealed two distinct classes of life cycle: those which transition between trypomastigotes, epimastigotes and/or amastigotes, and those which transition between promastigotes, choanomastigotes, opisthomastigote and/or amastigotes (Figure 2). These life cycle patterns clustered by both SSU and gGAPDH phylogeny (Figure 2A and D). On this basis we defined two morphological superclasses which we tentatively named for the apparent morphological distinction of whether the trypanosomatid has an extended region of lateral flagellum attachment; ‘juxtaform’ (from the Latin *juxta* (beside), incorporating trypomastigotes and epimastigotes), or a free flagellum with no lateral attachment extending beyond the flagellar pocket neck region no lateral attachment; ‘liberform’ (from the Latin *liber* (free), incorporating promastigotes, choanomastigotes and opisthomastigotes).

**Figure 3:**
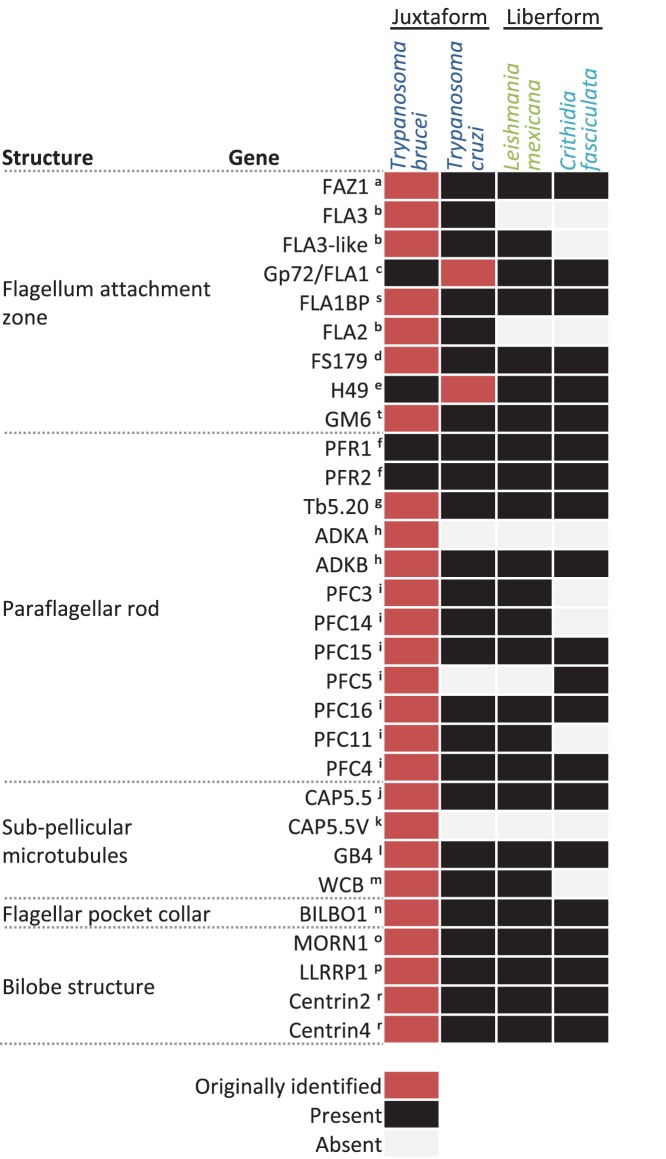
Conservation of known cytoskeletal-associated proteins between liberforms and juxtaforms. Summary of cytoskeleton-associated proteins, the organism in which they were originally identified (red), and possession (black) or absence (white) of a homolog, identified by reciprocal best BLASTp. PFR1 and 2 were originally identified in *Euglena gracilis*.^ a^ [222], ^b^ [223], ^c^ [4], ^d^ [224], ^e^ [225], ^f^ [226], ^g^ [227], ^h^ [228], ^i^ [229], ^j^ [230], ^k^ [231], ^l^ [232], ^m^ [233], ^n^ [234], ^o^ [235], ^p^ [236], ^r^ [237], ^s^ [238], ^t^ [239].

The differences in morphology of juxtaforms and liberforms suggests there may be significant differences in the presence or molecular composition of major cytoskeletal components, particularly the flagellum attachment zone (FAZ), but also the paraflagellar rod (PFR), sub-pellicular microtubules, the flagellar pocket collar and the bilobe structure. However, a survey of presence or absence of homologs to known proteins in these structures in two representative juxtaforms (*T. brucei* and *T. cruzi*) and two representative liberforms (*Leishmania mexicana* and *Crithidia fasciculata*) with published genomes [30]–[33] did not reveal any clear groups of absent homologs (Figure 3). Therefore, in the absence of clear molecular markers for these two morphological superclasses, species were assigned to each superclass on the basis of genera for analysis; *Trypanosoma* and *Blastocrithidia* being juxtaform and *Leishmania, Phytomonas, Leptomonas, Crithidia*, *Herpetomonas, Strigomonas, Angomonas* and *Paratrypanosoma* being liberform. Genera were taken as those listed by Sergei Podlipaev [23], or any later reclassification. We identified one species with an ambiguous superclass arising from reclassification of its genus over time; *Strigomonas culicis,* which was previously classified as a *Blastocrithidia* [34]. Unlike the other *Strigomonas* species, *S. culcis* was analysed with the juxtaforms because of its epimastigote morphology.

We analysed trypanosomatid shape and size diversity to determine whether limits to cell shape diversity across many trypanosomatid species correlate with different morphological classes, and what this implies for attainable trypanosomatid morphologies and their morphogenesis. *Trypanosoma* species have life cycle stages with juxtaform morphology both in and outside of the bloodstream where they may be subject to similar morphogenetic constraints, but different selective pressures. We therefore analysed the morphometric data particularly considering *Trypanosoma* species and whether there were limits to morphological diversity in different hosts which could be attributed to host selective pressures.

### Morphological Diversity of Juxtaform Trypanosomatids

**Figure 4:**
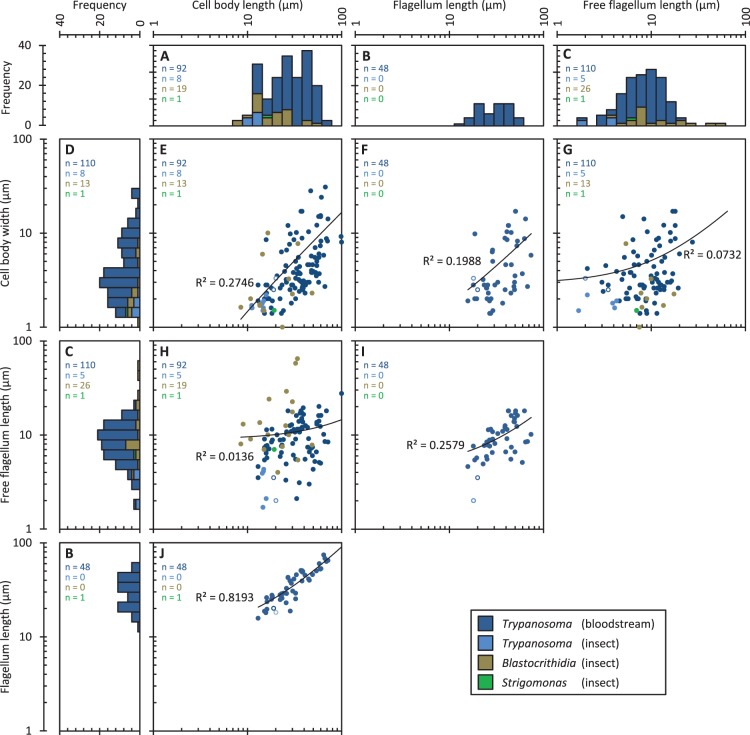
Diversity and correlation of morphological features in species with a juxtaform morphology. Data are colour coded by genus and host, *Trypanosoma* isolates are from either the vertebrate bloodstream of the insect host and all *Blastocrithidia* and *Strigomonas* isolates are from the insect host. ***n*** numbers for each plot are indicated in the top left. Solid black lines indicate the linear regression fit line for bloodstream *Trypanosoma*; the regression coefficient (***R^2^***) is shown each plot. Axenic procyclic form *T. brucei* dimensions [39], [41] and bloodstream form dimensions are indicated with a open data point.

Our survey of trypanosomatid morphology from the bloodstream (see Materials and Methods) identified 110 isolates including examples from all vertebrate classes except reptiles and jawless fish. All isolates were *Trypanosoma* trypomastigotes. Trypanosomatid size and shape varied considerably; cell body length and width, free flagellum length and flagellum length each varied by at least a factor of 10 across species (Figure 4A to D, dark blue data series). Cell body width showed the greatest diversity (Figure 4D) and cell body length and flagellum length showed a similar distribution (Figure 4A and B). The smallest trypomastigotes were around 10 µm in length (Figure 4A), twice the typical maximum length of amastigotes of *L. mexicana*, *T. cruzi* and *Leptomonas oncopelti* [6], [35]–[37]. In order to assess if there were particular limits on diversity of trypomastigote cell shape, the correlations of these morphological features were analysed. In general there were only very weak correlations in parwise comparisons between free flagellum length, cell body width, cell body width and flagellum length for trypomastigotes (Figure 4E to J). Exceptions to this were cell body length and width, which showed a loose positive correlation (Figure 4E), and cell body length and flagellum length, which showed a strong positive correlation (Figure 4J). Linear regression for each plot indicated a significantly non-zero gradient (linear regression gradient test, 5% significance), indicating a weak tendency for larger trypanosomes to be larger in every respect. The lack of strong correlations of any morphological measure, with the exception that flagellum length appears to be linked with cell body length, indicates that the range of cell shapes that can be attained is large.

Insect-inhabiting juxtaform species were all members of either the *Trypanosoma* or *Blastocrithidia* genus. Our survey of trypanosomatids with a laterally attached flagellum from insect hosts yielded comparatively few (34) quantitative descriptions of morphology, and were dominated by epimastigotes of *Blastocrithidia* (25) and *Strigomonas* (1) species. Despite the large number of known *Trypanosoma* species which infect terrestrial animals, all expected to have a life cycle stage in an insect host, few morphological descriptions of *Trypanosoma* met our inclusion criteria. *Trypanosoma* are therefore underrepresented in this data set. Kinetoplast-posterior distance was not reported in any case which prevented analysis of total flagellum length. We analysed the morphology of these isolates to determine whether the diversity of trypomastigote morphologies seen in the bloodstream could also occur in juxtaform morphologies outside the bloodstream environment.

The range of cell body length, cell body width and free flagellum lengths reported in juxtaform isolates from the insect host were large (Figure 4A to D, light blue, brown and green data series), with a range approaching that of bloodstream-inhabiting trypomastigotes. The distribution of cell body length and width were significantly skewed towards smaller dimensions relative to trypomastigotes in the bloodstream (KS test, 5% significance) while there was no significant difference in the distribution of free flagellum length (KS test, 5% significance). Correlative analysis to assess morphological diversity in detail was not possible due to the small number of isolates, however the dimensions of parasites within these isolates was similar to that of small trypomastigotes (Figure 4E to J).

### Morphological Diversity of Liberform Trypanosomatids

Many trypanosomatids inhabit invertebrate hosts, often within the alimentary tract and associated organs like the salivary glands. Promastigotes, choanomastigotes and opisthomastigotes (liberforms) are common in these environments, however epimastigotes and trypomastigotes (juxtaforms) and amastigotes are also widely reported. Our analysis of liberform morphology in the invertebrate host yielded quantitative morphological data from 103 isolates (see Materials and Methods), all of which were from insect hosts. Morphology diversity within these isolates was analysed to determine which morphological trends and limits observed within liberforms occur in juxtaforms, and *vice versa*.

**Figure 5:**
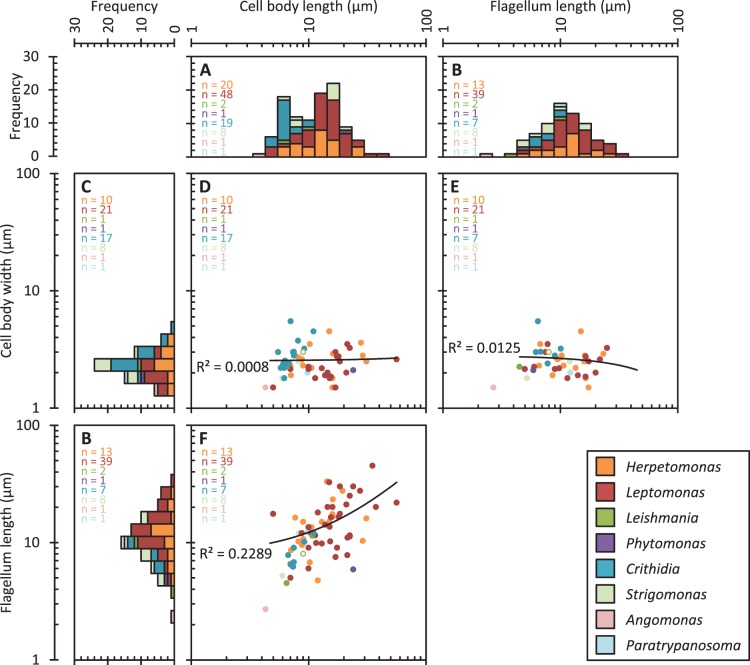
Diversity and correlation of morphological features in species with a liberform morphology. Data are colour coded by genus, all isolates are derived from the insect host. ***n*** numbers for each plot are indicated in the top left. Solid black lines indicate the linear regression fit line of data for all genera combined; the regression coefficient (***R^2^***) is shown each plot. Axenic promastigote *L. mexicana* dimensions [38] are indicated with an open data point.

Our survey of liberforms from insect hosts showed that both cell body and flagellum length vary by a factor of 10 (Figure 5A and B), a similar range to juxtaforms (Figure 4A to C). Cell width, however, only varied by a factor of approximately 3, with a maximum width of 4.5 µm reported (Figure 5C). This was unlike the large diversity of width of both trypomastigotes in the bloodstream and epimastigotes in the insect host (Figure 4D). The smallest liberforms, with several around 4 µm in length, were similar in size to amastigotes. Within the liberform superclass there was no significant difference between the distribution of cell body length, width and flagellum of the best-represented genera, *Herpetomonas* and *Leptomonas* (KS test, 5% significance). In contrast *Crithidia*, the third-best represented genus, had significantly shorter and wider cell bodies, and a shorter flagellum (KS test, 5% significance). The overall distribution of cell body length and width in liberforms were significantly skewed towards smaller dimensions relative to trypomastigotes in the bloodstream (KS test, 5% significance). The morphology of the early-branching *Paratrypanosoma confusum* [26] was not notable among the liberforms; all measured parameters were in the middle of the range (Figure 5A–F).

Limitations on cell shape diversity for liberforms were analysed through correlation of morphological measures. There was no strong correlation of cell body length, flagellum length or cell body width (Figure 5D to F). Linear regression for each plot indicated a significantly non-zero gradient (linear regression gradient test, 5% significance) only for the correlation of cell body length with free flagellum length (Figure 5F). This differed from cell body width in trypomastigotes in the bloodstream, which showed large diversity (Figure 4D) and a weak correlation with other morphological measures (Figure 4E to G). There therefore appears to be an additional constraint for a narrow cell width of liberforms in the invertebrate host, maintained across trypanosomatids inhabiting the full range of insect hosts. Of the 15 epimastigote isolates from invertebrates with width data available, 27% had widths greater than 4.5 µm (Figure 4D); this is wider than the largest width reported for liberforms. This suggests the constraint of liberform cells to a narrow range of widths may be intrinsic to cell organisation within this superclass, as juxtaforms (specifically epimastigotes) in invertebrate gut environments do not experience the same constraint. This limit on width is unlikely to be the only limit to morphological diversity, other selection-associated morphological adaptations associated with particular host properties are also likely.

### Morphological Diversity within Isolates

In addition to recording the average morphological properties of isolates, the range of morphological variation *within* an isolate was also recorded for our meta-analysis. Within any individual isolate some degree of morphological variation arising from different life cycle and cell cycle stages would be anticipated, and this diversity provides a means to gain insight into the processes that may be involved.

**Figure 6:**
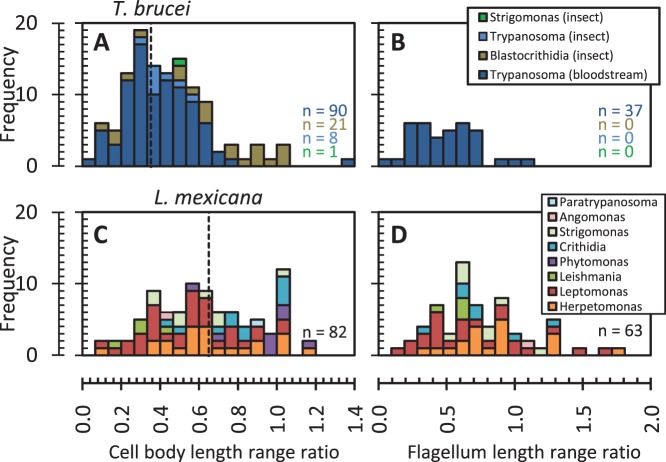
Range of cell body and flagellum length within isolates of juxtaforms and liberforms. Histograms of the ratio of the cell body length range within an isolate to the mean (the cell body ‘length range ratio’) (**A** and **C**) and flagellum length range ratio (**B** and **D**) for juxtaforms (**A** and **B**) and liberforms (**C** and **D**). ***n*** numbers for each plot are indicated in the bottom right. Vertical dashed line indicates where either proliferating axenic procyclic *T. brucei* trypomastigotes [39], [40] or *L. mexicana* promastigotes [38] would be positioned on the cell body length plots.

We quantified the range of cell body and flagellum length variation by determining the ratio of the range to the mean, a dimensionless value we call the ‘length range ratio’. The cell body length range ratio varied greatly between isolates, ranging from near 0.0 (no range in length) to over 1.0 (Figure 6). The average cell body length range ratio in liberforms in the insect host was 0.74±0.51, significantly larger (Student’s t-test, 5% significance level) and with a wider distribution than trypomastigotes in both the insect and the bloodstream, 0.41±0.19 (Figure 6A and C). The mean length range ratio in these two classes were consistent with those that may be expected from the cell cycle, based on axenic promastigote *L. mexicana* where the length range ratio is 0.67 [38] and trypomastigote procyclic *T. brucei* where the length range ratio is 0.35 [39], [40]. The average cell body length range ratio of epimastigotes (*Blastocrithidia* species) was 0.73±0.37, similar to the liberforms and significantly larger (Student’s t-test, 5% significance) than trypomastigotes in the bloodstream (Figure 6A). Whether a large range in cell body length is associated with the host environment (for example reflecting more life cycle substages within the insect host), or is associated with an intrinsic aspect of cell organisation cannot be determined without more extensive data from trypomastigotes in the insect environment.

Flagellum length showed a similar diversity to cell body length within isolates, with a mean flagellum length range ratio of 0.90±0.75 in liberforms in the insect host, significantly larger (Student’s t-test, 5% significance level) and with a wider distribution than trypomastigotes in the bloodstream, 0.51±0.25 (Figure 6B and D). This is consistent with the observed correlation of flagellum length with cell body length in trypomastigotes (Figure 4J), but not liberforms (Figure 5F), and the possible link of flagellum length with cell body length in juxtaforms. This is broadly consistent with the ranges of flagellum length which occur during axenic promastigote *L. mexicana* growth, in which a wide range of flagellar lengths are observed [38], and trypomastigote procyclic *T. brucei* division, where the flagellum length is less variable [41].

### Extrinsic Limits on Trypomastigote Diversity in the Bloodstream

Having analysed whether there are limits on trypanosomatid morphological diversity which seem associated with the liberform or juxtaform morphological superclasses (presence or absence of a laterally attached flagellum), we can now address whether additional limits on cell shape occur associated with selective pressures. Selective pressures on trypanosomatid morphology would be expected to manifest as limits on shape diversity which do not correlate with morphological class, but instead with the host environment and its properties.

**Figure 7:**
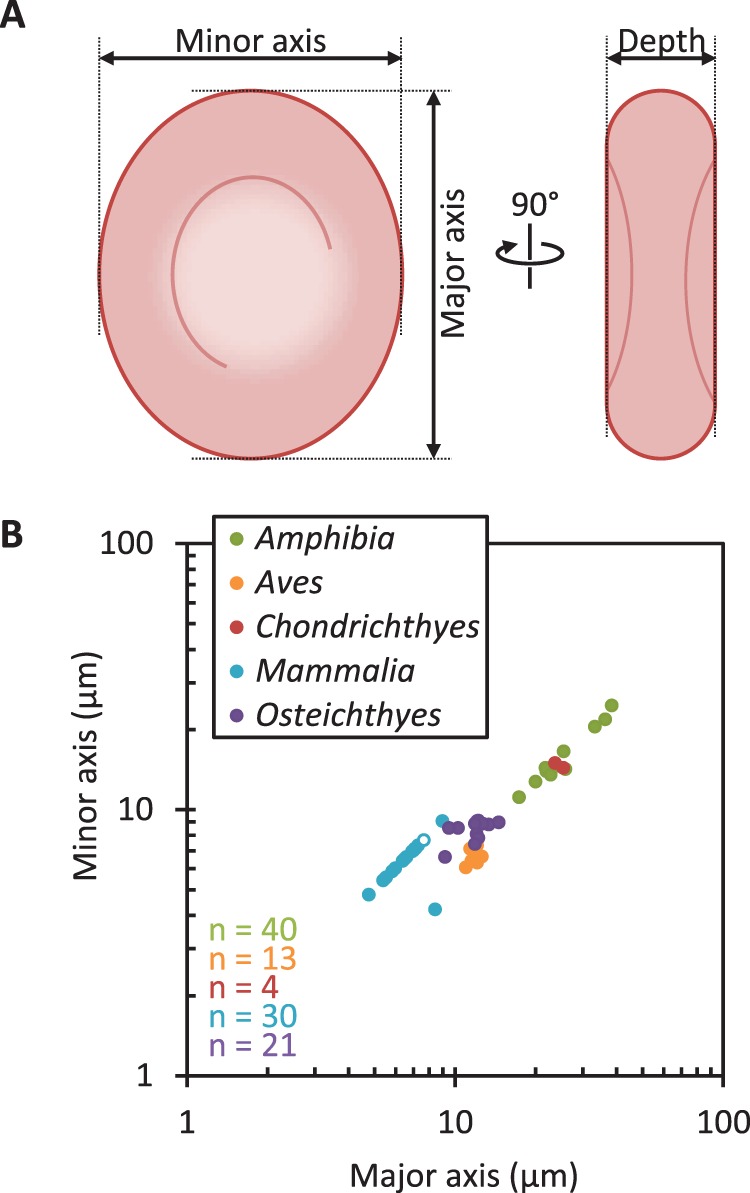
Diversity of erythrocyte dimensions of hosts from which bloodstream *Trypanosoma* isolates were derived. **A.** Diagram of erythrocyte measurements used to characterise their shape. **B.** Correlation of erythrocyte major axis and minor axis from vertebrate hosts of *Trypanosoma* parasites, subcategorised by class. ***n*** numbers for each genus are indicated in the bottom left. *Homo sapiens* erythrocyte dimensions are indicated with an open data point.

In this context *Trypanosoma* is notable as the only genus which inhabits the vertebrate bloodstream; it is also the only genus in which the trypomastigote morphology occurs. Furthermore it is the only genus defined by a unique morphological class, the trypomastigote, where the genus has been shown to be monophyletic by modern molecular phylogeny [25]. While *Trypanosoma* species can attain an epimastigote or amastigote morphology at some life cycle stages, they are almost universally found as a trypomastigote in the bloodstream environment. *Trypanosoma lewisi* is an exception to this where epimastigotes can be found in peripheral tissues, however even in this case parasites in the blood during high parasitaemias are trypomastigote [42]. This strong association of trypomastigote morphology with life in the bloodstream suggests there may also be associated selection pressures on trypomastigote size and shape; for example it has been suggested that *T. brucei* is of the correct size to allow effective motility amongst human erythrocytes [43]. The physical properties of a bloodstream environment are linked with the dimensions of host erythrocytes [44], therefore we aimed to detect selective pressures on trypomastigotes dimensions as limits on trypomastigote size diversity leading to a correlation of trypomastigote dimensions with host erythrocyte dimensions. Erythrocytes size is described by the dimensions of the major and minor axis of the elliptical face (Figure 7A) and, in the host species surveyed, these varied by a factor of 10 (Figure 7B), similar to the range of trypomastigote dimensions.

**Figure 8:**
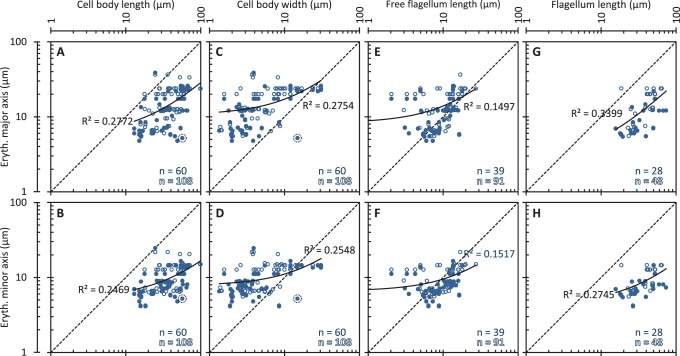
Limits on bloodstream-inhabiting trypanosomatid morphology relative to host erythrocyte dimensions. Correlations of trypanosomatid cell body length, cell body width, free flagellum length and flagellum length with host erythrocyte major axis and minor axis. Solid data points indicate host species-specific erythrocyte data, where available, open data points indicate the average of erythrocyte data available for the host genus. Solid lines indicate the linear regression fit line for genus data; the regression coefficient (***R^2^***) is shown on each plot. Dashed lines indicate the line along which trypanosomatid and host erythrocyte dimensions would be equal. *T. binneyi* data points are circled with a dotted line. ***n*** numbers for each plot are indicated in the bottom right.

Host erythrocyte major and minor axis did not show a clear correlation with trypanosomatid cell body length, width or free flagellum length (Figure 8). While there was little direct correlation other trends emerged: 95% of trypomastigotes were narrower than the host erythrocyte major axis (Figure 8C). Even large mammalian-infective *Trypanosoma* species, such as members of the monophyletic subgenus *Megatrypanum*, *T. (M.) cervi* and *T. (M.) theileri* [45], adhered to this width constraint. Using the test criterion of the number of trypanosomatids with a width smaller than the host erythrocyte major axis, a Monte Carlo permutation test indicated this trend was significant (5% significance level). 98% of trypomastigotes were also longer than the host erythrocyte major axis (Figure 8A) and all are longer than the minor axis (Figure 8B). Flagellum length, which correlates with cell body length (Figure 4J), showed a similar trend (Figure 8G and H). These two trends were, however, not significant by a Monte Carlo permutation test (5% significance level). Lack of significance likely arises from the large minimum trypomastigote cell length of approximately 10 µm.

One species which deviated from the apparent constraint on width by a large degree was identified. *Trypanosoma binneyi* has a large trypomastigote bloodstream form (47 to 67 µm long, 11 to 15 µm wide [46]) and can inhabit the bloodstream of the platypus (*Ornithorhynchus anatinus*) which has small erythrocytes similar to other mammals (5.2 µm diameter [47]). *T. binneyi* is a member of the fish and amphibian *Trypanosoma* clade [48], [49] and the platypus inhabits an aquatic environment. There is therefore the possibility that the platypus is not a normal host or is not the major host of this species, or that the platypus has only recently become a transmissive host of *T. binneyi*.

**Figure 9:**
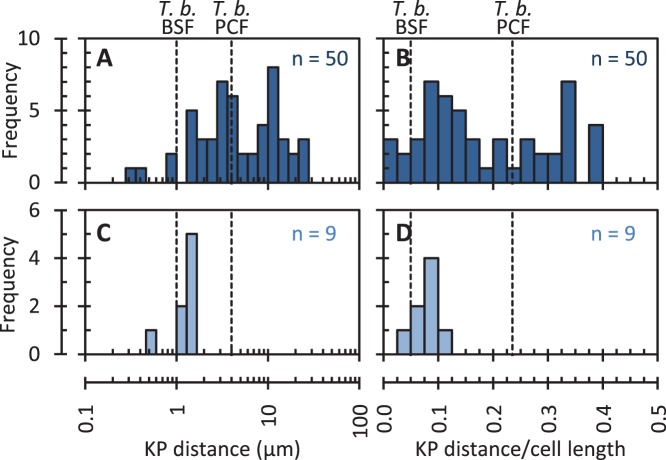
Limits on kinetoplast position in bloodstream-inhabiting trypanosomatid species. Histograms of kinetoplast-posterior (KP) distance (**A** and **C**) and the ratio of KP distance to cell body length (**B** and **D**) for all trypanosomatid isolates from the bloodstream (**A** and **B**) and a separate set of only mammalian-infective African trypanosomes (**C** and **D**). ***n*** numbers for each plot are indicated in the top right. Vertical dashed lines indicate where axenic PCF and BSF *T. brucei* would be positioned on these plots.

The universality of the trypomastigote (as opposed to epimastigote) morphology in the blood also supports the idea that a short kinetoplast posterior distance may be an adaptation for the bloodstream. As the nucleus is typically positioned near the centre of a trypomastigote the kinetoplast would be expected to be in the posterior half of the cell, however histograms of kinetoplast distance from the posterior (Figure 9A) and kinetoplast distance from the posterior as a fraction of cell body length (Figure 9B) for *Trypanosoma* spp. revealed no trend for kinetoplast/flagellar pocket positioning particularly close to the posterior of trypomastigotes in the bloodstream. Few trypomastigotes had a kinetoplast very close to the posterior, as observed in bloodstream form *T. brucei*, and many species had a kinetoplast positioned far from the posterior, often further than for procyclic form *T. brucei*. No particular bias of kinetoplast position between different host classes could be identified. While a short posterior–kinetoplast distance was not a feature of all trypomastigotes in the bloodstream, the kinetoplast is positioned less than 2 µm (≤10% cell body length) from the posterior in the bloodstream forms of several mammalian-infective African trypanosome species [50]–[57] (Figure 9C and D). Based on molecular phylogeny of 4 of these species (*T. brucei, T. vivax*, *T. simiae* and *T. congolense*), and the status of *T. evansi* as a petite mutant of *T. brucei* [58], these are members of the monophyletic *T. brucei* (Salivarian) clade [59], [60].

## Discussion

We have performed a meta-analysis of the morphologies of motile life cycle stages of a wide range of trypanosomatid parasites derived from two major environments they inhabit; the invertebrate gut and the vertebrate bloodstream. For analysis these were placed into two morphological superclasses, the liberforms and juxtaforms, defined from genera which can attain the promastigote, choanamastigote, opisthomastigote or amastigote morphology (*Leishmania, Phytomonas, Herpetomonas, Leptomonas, Crithidia, Angomonas, Strigomonas and Paratrypanosoma*) and the trypomastigote, epimastigote or amastigote morphology (*Trypanosoma* and *Blastocrithidia*) respectively. *Strigomonas* was the only genus where current classification indicates both juxtaforms (with one example, *S. culcis*) and liberforms (other species) occur [26]. We propose that these two morphological superclasses represent biologically meaningful groups. The fact that we could find no species which has the capacity to transition between morphologies unique to each of these superclasses through life cycle differentiation events (Figure 2B) supports this view. This was further supported by the clustering of these life cycle patterns by phylogeny inferred from SSU rRNA and gGAPDH gene sequences (Figure 2A and D), although juxtaform monoxenous insect trypanosomatids (*Blastocrithidia* and a subset of *Strigomonas* species) are currently poorly sampled.

Limits on diversity were analysed through the morphological parameters of cell body length and width, free and total flagellum length and kinetoplast position. Morphology of internal structures, such as the kinetoplast, were not considered in this analysis. The morphological parameters we analysed typically varied over a 10-fold range (Figure 4A to D and 5A to C). Within this range several patterns in morphological diversity of the trypanosomatids in different environments were identified. Firstly, juxtaforms and liberforms had different limits on cell shape diversity: the flagellum length of trypomastigotes, whilst in the bloodstream, was limited to a similar length as the cell body (Figure 4J) while in liberforms, whilst in an insect host, this was not the case (Figure 5G). Conversely, the cell body width in liberforms was limited to a narrow range (Figure 5D and E) while in juxtaforms it was not. In fact in trypomastigotes there was a loose positive correlation with cell size (Figure 2E). Juxtaforms were also limited to a larger minimum cell length, approximately 10 µm, (Figure 4A) than liberforms (Figure 5A).

Of the traditional kinetoplastid morphological classes (Figure 1) the trypomastigote is a notable juxtaform as it occurs near universally (we identified one exception, *T. lewisi* [42]) in vertebrate bloodstream-inhabiting life cycle stages, representing a near perfect coincidence of form and environment. Trypomastigotes in the bloodstream showed a trend for widths narrower than the minor axis of the host erythrocytes (Figure 8C). They also had lengths longer than the major axis of the host erythrocytes (Figure 8A), which may be associated with the limit on minimum cell length in morphologies with an attached flagellum (Figure 4A). Finally the flagellar pocket of the Salivarian clade of African mammalian-infective trypanosomes was located close to the posterior end of the cell, although in more diverse trypomastigotes this was not consistently observed.

We suggest these limits on morphology diversity can be explained through three groups of biological features; the physical properties of the host or vector environment, the mechanisms of cell morphogenesis through the cell cycle and, more speculatively, some functions of the flagellum in the host. The second of these is an intrinsic constraint of cell organisation, while the first and third represent extrinsic selective pressures from a host.

### Extrinsic Morphological Constraints from the Host

Trypanosomatids need to fit through the capillaries of the host, the diameters of which average 80% of the largest erythrocyte dimension for vertebrate species [44]. The high mechanical rigidity of microtubules [61] in the sub-pellicular array would be expected to limit the degree to which trypanosomatid cells can deform to pass through the capillaries, restricting the range of morphologies which would be able to do so. The limited range of trypanosomatid widths (Figure 8C) is consistent with the idea that this mechanical effect exerts a selective pressure. It has previously been shown that the flagellum wavelength, cell body length and rotation of *T. brucei* while swimming confers a mechanical advantage when swimming amongst objects the size of human erythrocytes [43]. It is possible that the limit on minimum trypomastigote cell length (Figure 4A and 8G and H) indicates that this mechanism provides a similar advantage for motility of other species in their respective vertebrate hosts, although juxtaforms in the insect host shared this limit on minimum cell length.

Blood flow velocity in mammalian capillaries [62] is at least one order of magnitude higher than maximum *T. brucei* bloodstream form swimming speed [63], [64] making the role of swimming unclear as it is too slow to overcome the bulk flow of the blood. Nonetheless bloodstream form trypanosomes are typically highly active swimmers, and this has been confirmed in detail *in vivo* [65]. The function of this motility is debated, and there are many hypotheses including a role in tissue penetration (such as establishment of neurological stages of African sleeping sickness) or cell penetration (as for establishment of intracellular life cycle stages of *Trypanosoma cruzi* [66]). A moving cell body may also help escape from low affinity interaction with phagocytes, and avoid uptake. These hypotheses do not entail any particular requirement for a trypomastigote morphology, or any other specific morphological feature, for their function, and could therefore not be tested by analysing the observed range of trypomastigote morphologies.

In contrast, the proposed function of hydrodynamic surface clearance does suggest a particular advantageous morphology. It has been shown that hydrodynamic flow from swimming assists clearance of surface bound antibodies, and may assist avoidance of the adaptive immune response [67], [68]. Hydrodynamic flow forces arising from swimming concentrates large surface molecules, including bound immunoglobulins, at the posterior end of the cell. As endocytosis occurs exclusively within the flagellar pocket and trypanosomatids swim in the flagellum-first direction this suggests a trypomastigote morphology and a flagellar pocket located close to the posterior end would confer a selective advantage through an improved clearance rate [67], [68]. Our analysis indicated that the kinetoplast is consistently positioned near the posterior end in the *T. brucei* (Salivarian) clade (Figure 9). Members of this clade have a thick surface coat in the bloodstream [69], [70], and undergo antigenic variation *via* their monotypic coat of GPI-anchored variable surface glycoproteins (VSG) [71], [72]. The *T. brucei* VSG coat is internalised and recycled by an exceptionally high rate of endocytosis [73] and recycling of VSG with bound immunoglobulins is assisted by hydrodynamic effects [68]. The selective pressure would therefore be reduced antigenicity of the monotypic surface coat. While it appears this mechanism may have applied a selective pressure in the Salivarian clade it is not a general phenomenon amongst trypomastigotes; little constraint on kinetoplast location was identified in other species (Figure 9) so other methods of immune evasion may be dominant in those cases.

### Intrinsic Morphological Constraints Associated with Morphogenesis

There are differences in the mechanisms of morphogenesis of different trypanosomatid morphologies, particularly in the regulation of cell body length, which may give rise to differing constraints on parasite morphology. In trypomastigote *T. brucei,* cell body length depends on flagellum length. Mutations which affect growth or attachment of the flagellum often give rise to abnormally short daughter cells [39], [74]–[76], and this mechanism appears to be used to cause morphological change during life cycle progression [77]. In contrast promastigote *L. mexicana* mutants with elongated or shortened flagella do not experience any large change to cell length or problems with division [78]–[80]. The differing degrees of correlation of cell body and flagellum length of juxtaforms and liberforms (Figure 4 and 5) suggest the morphogenesis of *T. brucei* and *L. mexicana* are representative of these two superclasses respectively, with lateral flagellum attachment and flagellum length mediating control of cell length in juxtaforms but not in liberforms. It seems plausible the greater minimum cell length observed in trypomastigotes is a morphogenetic constraint associated with this mechanism of cell length control, however further analysis of length regulation and morphogenesis in epimastigotes through the cell cycle will be required to confirm these hypotheses.

**Figure 10:**
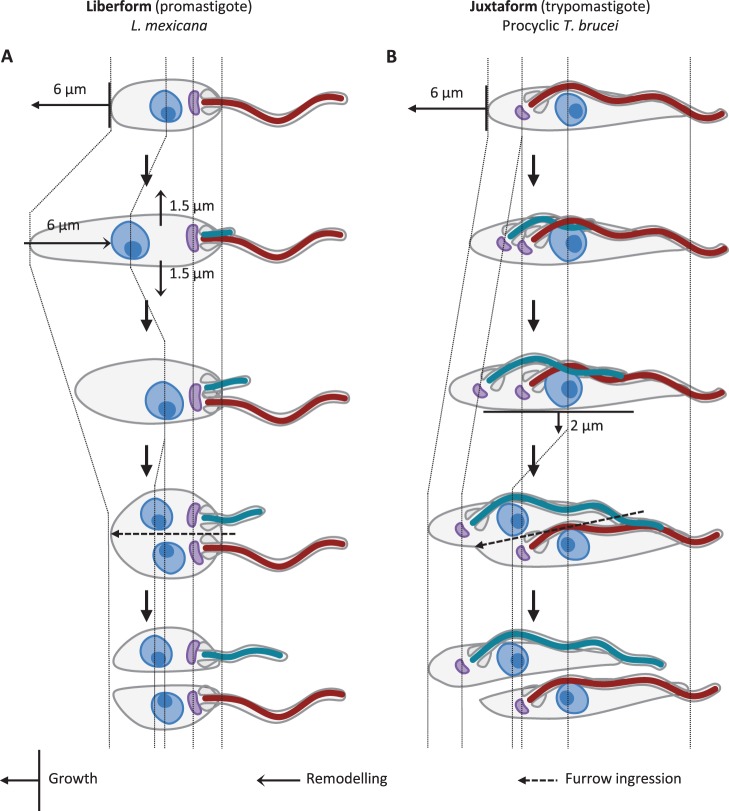
Cell body morphogenesis of a promastigote and trypomastigote through the cell cycle. Cell growth is indicated by arrows with a perpendicular bar, the bar indicates the region that grows. Cell remodelling is indicated by barb-headed arrows. The path of furrow ingression is indicated by dashed arrows. The old and new flagellum are coloured red and turquoise respectively. **A.** Summary of the morphological changes of an example liberform (promastigote *L. mexicana*) through the cell cycle [38]. The cell grows in length, then remodels the cytoskeleton during cytokinesis. **B.** Summary of the morphological changes of an example juxtaform (trypomastigote procyclic *T. brucei*) through the cell cycle [39], [81], [240]. The cell first grows in length then in width prior to cytokinesis.

Unlike flagellum and cell body length, cell body width in liberforms seemed subject to an intrinsic constraint, limiting cells to a narrow range of widths (Figure 5), a constraint not seen in juxtaforms (Figure 4). This limit to morphological diversity was particularly striking and may be associated with the mechanisms used for cell body growth. Cell body morphogenesis in trypanosomatids is linked with the growth of the sub-pellicular microtubule array, which maintains a uniform coverage of equally spaced microtubules under the plasma membrane at all stages of the cell and life cycles. At least two mechanisms of array growth appear to occur, based on the identification of non-detyrosinated tubulin of newly polymerised microtubules in *T. brucei* [81]. The array can increase in length by polymerisation of a group of microtubules in a coordinated manner; this occurs at the posterior end of *T. brucei* and *L. mexicana* may undergo a similar growth at the posterior end [38], [82]. New microtubules can also be inserted into the existing array, leading to an increased width of that section of the cytoskeleton; this is thought to occur throughout the mid portion of the cell body during later stages of division in *T. brucei* [81] (Figure 10B). Steady growth in width is not a morphogenetic process used by *L. mexicana,* which instead undergoes a rapid remodelling in shape with an increase in width and decrease in length in the lead up to cytokinesis [38] (Figure 10A).

This quantitative analysis of mechanisms used for morphogenesis of *T. brucei* procyclic trypomastigotes and *L. mexicana* promastigotes (Figure 10), combined with the apparent constraint on cell widths attained by liberforms (Figure 5D and E), may indicate it is a general phenomenon that liberforms use rapid cellular remodelling instead of gradual growth for morphogenesis; it may be the case that the second method of array growth, increase in width through microtubule insertion, does not occur in this superclass. Conversely the lateral attachment of the flagellum in trypomastigotes may prevent the dramatic rearrangement of cell shape used by *Leishmania* for cytokinesis and enforce a different mechanism for increasing in width. Again analysis of the morphogenesis of epimastigotes through the cell cycle will be required to test if this is a universal feature of juxtaform morphogenesis. Existing qualitative descriptions and diagrams of dividing liberform trypanosomatids, particularly *Leptomonas* spp. [83], [84], and juxtaform trypanosomatids, particularly mammalian-infective trypanosomes [22] also support this hypothesis. Furthermore the range of cell body and flagellum lengths seen within isolates is broadly consistent with morphological variation as part of a cell cycle similar to *L. mexicana* or *T. brucei* for liberforms and juxtaforms respectively (Figure 6). Together, and in combination with apparent difference in role of the flagellum in cell body length regulation, these further support the concept of the liberform and juxtaform morphological superclasses and that differences in their morphogenesis may impose different constraints on cell shape.

There has been growing evidence that definition of trypanosomatid genera by the morphological classes they attain in the life cycle does not reflect the molecular phylogeny of these species, and there have since been efforts to regroup the species into monophyletic subfamilies on the basis of genetic analyses [25]. The recently-identified promastigote *Paratrypanosoma confusum* strongly suggests the ancestral morphology of trypanosomatids was liberform [26], implying the juxtaform morphology has arisen in at least one well-sampled lineage (*Trypanosoma*) and two less well-sampled lineages (*Blastocrithida* and *Strigomonas*). We suggest that morphological data still has biological value in this context; *Trypanosoma* are universally juxtaform and unique in their capacity to live in the vertebrate bloodstream, and the superclassification of trypanosomatid morphologies into the liberform and juxtaform superclasses correlates well with the ability for parasite morphological change through the life cycle (Figure 2) and capacity for morphological diversity (Figure 4 and 5), independent of whether juxtaforms or liberforms prove to be mono- or paraphyletic. Our analysis showed that protein components of cytoskeletal structures particularly associated with juxtaforms or liberforms respectively, have not been lost or gained in a modular fashion. The FAZ being the clearest example of this. Clearly identifiable homologues of the majority of genes encoding such proteins are present in members of the other superclass (Figure 3). This suggests that the mechanisms that underlie morphological differences are better interpreted as modulation of the cytoskeleton rather than modular loss or gain of the capacity to form particular cytoskeletal structures, like the FAZ. It may therefore be unsurprising that multiple lineages seem to have independently evolved a juxtaform morphology.

The amastigote morphology, which is poorly defined, occurs in proliferative intracellular *Leishmania* and *Trypanosoma* [10]–[12], encysted life cycle stages of monoxenous insect parasites [14] and occasionally other forms. It cannot be directly classified as juxtaform or liberform as it lacks a long external flagellum. One prediction of the superclass model is the possibility that juxtaform and liberform amastigotes may be heavily modulated examples of epimastigotes and promastigotes respectively; there may be morphological differences between the juxtaform and liberform amastigote in the structure of the flagellum exit from the pocket, the ultrastructure of the collar and neck of the pocket, and presence or absence of an elongated FAZ-like structure.

### The Function of the Flagellum

The classic morphological classes and our proposed juxtaform and liberform superclasses are all defined on the basis of the flagellum, and as a result many of these discussion points touch aspects of flagellum function in trypanosomatids. It is valuable to consider their implications for the broader question of why does any trypanosomatid have a particular morphology at any particular life cycle stage, and what is the function of the flagellum within that morphology. The flagellum, which is universally present in trypanosomatids, is a central component in the vital flagellar pocket structure; it is the flagellum and its interaction with the flagellar pocket collar which defines the pocket structure and orientation [85]. Endocytosis (and the associated processes of receptor mediated uptake, surface recycling and antibody clearance) occur only in this membrane domain therefore it appears the minimal trypanosomatid cell requires at least a short flagellum to maintain this structure. This is consistent with the presence of very short flagella in amastigotes, whether living inside the cytoplasm (like *T. cruzi*), a parasitophorous vacuole (like *Leishmania*) or extracellularly (like the monoxenous insect trypanosomatids) [21], [86]. In addition to its role in the formation of the flagellar pocket, the trypanosomatid flagellum has at least four other major functions: surface attachment, motility, control of morphogenesis (as described above) and sensation (for which there is still limited evidence).

Surface attachment in the insect is common in most trypanosomatids [28] and is usually mediated *via* the flagellum. Attachment is associated with formation of hemidesmosome-like structures which have been described in detail in diverse genera; *Crithidia* [87], *Leptomonas* [88], *Trypanosoma* [89]–[93] and *Leishmania* [94]–[96]. These descriptions all concern epimastigotes or promastigotes. While the trypomastigote flagellum is capable of adhesion to a surface (its own cell body *via* the FAZ [97]) there are no known examples of trypomastigotes adhering to external surfaces, such as a fly intestine. In the non-trypomastigote morphologies, the ability to attach to surfaces may be a vital and limiting role of the extended free flagellum. Further analysis will be required to determine what constraints on morphology have arisen from selective pressures exerted by the invertebrate host.

The universality of the trypomastigote morphology and its motile flagellum in the bloodstream suggest a vital role of this morphology, which we speculate may be primarily associated with the motility of the flagellum. While trypomastigote life cycle stages do occur outside of the blood (for example the *T. brucei* procyclic (proliferative) and metacyclic (non-proliferative) forms [1], [98]) they are comparatively rare in the vector. Instead *Trypanosoma* spp. often have an epimastigote morphology, like *Blastocrithidia*, in the insect. It is therefore tempting to speculate that the trypomastigote morphology is a specialised juxtaform morphology, evolution of which was a key event in adaptation to the bloodstream environment by an ancestral monoxenous insect parasite. Within the bloodstream we have shown there are limits on kinetoplast position, cell body width and cell body and flagellum length which appear linked with plausible selective pressures associated with surface coat recycling (consistent with a motility role of the flagellum) and the size of host capillaries. Furthermore there appear to be particular constraints on cell morphogenesis, consistent with a morphogenetic role of the flagellum, associated specifically with the juxtaform cell shape. These findings give clear insights into functions of the typomastigote cell shape and its long flagellum. With the ability to generate morphological mutants in the laboratory and to compare genomes of diverse trypanosomatids, it is now feasible to search for the underlying principles that shape the trypanosomatid cell.

## Materials and Methods

Literature concerning identification of new species and surveys of prevalence of trypanosomatid infections was systematically surveyed from the literature. Cell body length, cell body width, free flagellum length and kinetoplast to posterior distance were recorded, but morphological data was only included if certain criteria were met:

For bloodstream*-*inhabiting species, trypanosomatid dimensions from isolates were recorded if the parasite cell body length and width were available, host genus erythrocyte dimensions were available in the Erythrocyte Size Database [99] and if measurements were taken from blood isolates or axenic cultures immediately derived from isolates and if measurements were representative of the complete range of morphologies present. Erythrocyte major and minor axes were recorded for the host species (if available) in addition to the average erythrocyte major and minor axes across all species in the host genus. This generated a data set covering morphology of 110 isolates from the blood of 73 host vertebrates (covering 48 genera distributed across *Amphibia, Aves, Chondrichthyes, Mammalia* and *Osteichthyes*) (Table S1). *Trypanosoma* morphology references: [5], [47], [100]–[118], [118]–[140]. Original erythrocyte dimensions references: [47], [141]–[167].

*Trypanosoma* morphology data were supplemented with measurements of cell body length and kinetoplast-posterior distance from previously published illustrations (using ImageJ [168]) of the mammalian-infective African trypanosomes *T. brucei*, *T. evansi, T. vivax*, *T. simoae*, *T. congolense, T. nanum, T. pecorum* and *T. uniforme.* Original host species for these isolates was not clear, therefore these data were not included in the complete trypomastigote/erythrocyte morphology data set. Mammalian-infective African trypanosome references: [50]–[57].

For species inhabiting an invertebrate host, dimensions were recorded if parasite cell body and flagellum length were available, if measurements were taken from the initial isolate or axenic cultures immediately derived from isolates and if measurements were representative of the complete range of morphologies present. Only flagellated life cycle stages were included. This generated a data set covering morphology of 103 isolates of trypanosomatids with a free flagellum from 73 host insects (covering 63 genera) (Table S1) and a data set covering morphology of 35 isolates of trypanosomatids with a laterally attached flagellum from 27 host insects (covering 25 genera) (Table S1). *Crithidia, Leptomonas, Herpetomonas, Leishmania*, *Phytomonas, Angomonas, Strigomonas and Paratrypanosoma* references: [24], [26], [34], [83], [84], [169]–[201]. *Trypanosoma* and *Blastocrithidia* references: [24], [169], [170], [181], [182], [202], [203].

Mean, minimum, maximum and standard deviation of morphological measurements were recorded where available, where mean length was not provided it was derived from the mean of the minimum and maximum. Where minimum and maximum were not available they were estimated as the 5^th^ and 95^th^ percentiles (i.e. expected minimum and maximum for a sample of ***n*** = 20) from the mean and standard deviation where possible. Flagellum length of morphologies with a laterally attached flagellum was estimated from the sum of cell body length and free flagellum length, minus the kinetoplast-posterior distance. Minimum and maximum flagellum length were estimated using the root sum squared deviation of the minimum and maximum of cell body length, free flagellum length and kinetoplast-posterior distance from their respective means. All plots concerning trypanosomatid morphology are derived from these data and ***n*** numbers indicate the number of species for which the morphometric data required for the plot was available.

Trypanosomatid phylogeny was inferred from SSU and gGAPDH gene sequence data available in Genbank. Unless otherwise indicated sequences were identified by species name and when sequences from multiple clones or isolates were available a single representative sequence was selected by sequence length (complete gene sequences were preferable) and quality (no gaps or uncalled bases). Multiple sequence alignments were generated with ClustalW [204], Clustal Omega [205], Kalign [206], MAFFT [207], MUSCLE [208] and T-Coffee [209] then combined using MergeAlign [210] and the alignment trimmed to only columns with a MergeAlign score over 0.5. The phylogenetic tree was estimated by the neighbour joining method [211] with 1,000 bootstrap iterations. Nodes with less than 50% (0.50) bootstrap support were collapsed to polytomies, preserving branch length. Genbank accession numbers: SSU: M12676.1, FJ900241.2, AJ223566.1, AJ009140.1, AJ009143.1, GQ332360.1, X53913.1, M84225.1, U39577.1, Y00055.1, AF153037.2, L18872.1, DQ207573.1. gGAPDH: AJ620263.1, AF047493.1, DQ092548.1, EU084894.1, M26816.1, XM_001566870.1, XM_003877392.1, DQ092549.1, AJ620272.1, X52898.1, AJ620247.1, FJ968528.1, EU084900.1.

Presence and absence of homologs of proteins associated with cytoskeletal structures were identified by the criterion of reciprocal best BLASTp against the predicted protein sequences of a genome, with a minimum ***p***-value cutoff of 10^−5^. Analysis was performed online using TriTrypDB.org [30].

## Supporting Information

Table S1 **Morphometry of trypanosomatids.** The complete morphometric data set used for this analysis, derived from previously published data for many trypanosomatid species from different vertebrate and invertebrate hosts.
(XLSX)
